# Mindfulness training decreases the habituation response to persistent food stimulation

**DOI:** 10.1038/s41598-025-90172-3

**Published:** 2025-04-25

**Authors:** Alyssa Torske, Doris Schicker, Jessica Freiherr, Kathrin Koch

**Affiliations:** 1 School of Medicine and Health, Department of Diagnostic and Interventional Neuroradiology, Technical University of Munich, Munich, Germany; 2 TUM-Neuroimaging Center (TUM-NIC), Klinikum rechts der Isar, Technical University of Munich, Munich, Germany; 3 Sensory Analytics and Technologies, Fraunhofer Institute for Process Engineering and Packaging IVV, Freising, Germany; 4 Department of Psychiatry and Psychotherapy, Friedrich-Alexander-Universität Erlangen-Nürnberg, Erlangen, Germany

**Keywords:** Sensory perception, Habituation response, Mindfulness, Eating behavior, Neuroimaging, Feeding behaviour, Olfactory cortex, Perception, Sensory processing

## Abstract

Modern societies and their obesogenic environments expose individuals to persistent food stimulation. This frequent exposure can cause sensory systems to habituate or desensitize to food-related sensory stimulation. This can, in turn, lead to the reduction of pleasure associated with eating, which can elicit overeating behavior to attain the desired pleasurable effect. However, frequently engaging in overeating behavior can lead to excessive weight gain, which is associated with the development of metabolic and cardiovascular disease. Mindfulness training could serve as a tool to reduce the habituation response elicited by frequent food exposure while promoting mindful eating, emotion regulation, and reducing overeating behavior. To investigate this, the present study was registered as a clinical trial on the ISRCTN registry: trial ID ISRCTN12901054. In the study, meditation-naïve individuals with a tendency to stress-eat (*N* = 56) participated in either a 31-day, web-based, food-related mindfulness training or health training condition. Functional magnetic resonance imaging (fMRI) and behavioral data were acquired before and after the intervention. During the fMRI sessions, hungry and stressed participants were exposed to visual and olfactory high-calorie food stimuli. The results indicate that hunger and stress ratings increased in both groups during the fMRI sessions but that mindfulness training, in comparison to health training, may significantly reduce the habituation response to food stimuli. Our results demonstrate that the habituation response could be implicated through the increase of neural activity in brain regions involved in visual and olfactory processing as well as emotion regulation. This study, therefore, demonstrates that mindfulness training could improve the ability to attend to food stimuli, which may enhance the pleasurable experience of eating, thereby diminishing an individual’s tendency to engage in overeating behavior.

## Introduction

Sensory systems allow for the extraction of valuable information from the environment. The visual and olfactory systems, for example, can play an essential role in detecting and assessing the edibility, nutritional value, and safety of food^1,2^. Not only do sensory systems play an integral role in regulating perceived hunger, but stress has also been demonstrated to increase appetite and encourage eating behavior^3–8^. This serves as an adaptive strategy to ensure that individuals have enough energy to effectively respond to imminent threats in the environment^6,9^. While this response is advantageous from an evolutionary perspective, modern societies and their accompanying modern stressors introduce a mismatch between stress-induced appetite increases and the necessary energy requirements.

Not only do our modern, stressed societies inherently elicit an increase in appetite, but it is also important to consider the frequent food exposure produced by obesogenic environments. Therefore, when stressed individuals are persistently exposed to foods in obesogenic environments, significant appetite-enhancing effects are elicited. In fact, given this persistent exposure to food, the habituation response to food stimulation can occur^10,11^. The habituation response is thought to be an adaptive mechanism to prevent sensory overload and facilitate a focus on novel or potentially significant stimuli in the environment^10–12^. In obesogenic environments, the initial response to appealing food aromas or visuals may be strong, but with repeated exposure, sensory receptors adapt, leading to a decrease in perceived intensity^13,14^. Not only does the perception of the food stimuli decrease, but as an additional consequence, the rewarding nature and pleasurable experience associated with eating also diminish due to the decrease in the sensory perception of food^15^. This may, in turn, cause individuals to eat more (i.e. engage in overeating behavior) to compensate for the reduced satisfaction of eating elicited by the habituation response.

The cyclical nature of habituation-induced desensitization and subsequent overeating can lead to the development of unhealthy eating habits and may contribute to the overconsumption of food beyond the body’s actual physiological energy requirements. Recognizing this interplay between obesogenic environments, the habituation response, the stress response, and appetite increases highlights the importance of adopting strategies to manage stress and sensory perception. Breaking this cycle through the ability to reduce stress, increase emotion regulation, and increase attention could allow for the mitigation of overeating behavior, which is associated with an increase in caloric intake, excessive weight gain, and subsequent health-related consequences, such as cardiovascular and metabolic disease.

For weight management, mindful eating (for an overview of mindful eating see Mantzios)^16^ can provide an effective strategy (for a review see Artiles et al.)^17^ and influence reward anticipation in the midbrain^18^. However, the scientific understanding of mindful eating remains limited^19^. Additionally, a variety of mindfulness-based interventions can improve problematic eating behaviors (for a review see Warren et al.)^20^, though also here further research is needed^21^.

Mindfulness training, by fostering present-moment awareness and non-judgmental attention to sensory experiences^22^, could also be implemented as a tool to reduce the effects of the habituation response and stress-related overeating behavior. Mindfulness techniques encourage individuals to engage with the sensory aspects in a more intentional and conscious manner and could reduce stress-eating behavior^23,24^. Therefore, through mindfulness, individuals may develop a heightened awareness and sensitivity to the flavors, textures, and aromas of food, thereby disrupting the habituation process that often leads to diminished responsiveness and reward value to these sensory stimuli.

This increase in awareness, fostered by mindfulness training, could restore a sense of novelty to the eating experience, allowing each bite to be more pleasurable and satisfying, ultimately reducing the likelihood of overeating to compensate for reduced pleasure elicited by the habituation response^25^. Additionally, mindfulness practices promote enhanced interoceptive awareness including perceived hunger and fullness cues, allowing individuals to recognize and respond to their body’s signals more effectively^25–27^. Not only can mindfulness training play a role in the perception of food stimuli, but it could also increase stress sensitivity, rendering individuals to reduce their stress response^28–31^. Mindfulness not only has the ability to cultivate interoceptive awareness, but it can also play a role in increasing emotion regulation, which in turn can reduce stress sensitivity and consequently affect eating behavior^26,27,32–36^. Consequently, mindfulness training could be very effective in inhibiting stress-related overeating behavior as well as the overeating behavior generated by the habituation response, all while fostering a more mindful relationship with food.

To study the effects of mindfulness training on the sensory habituation response pertaining to food stimulation, functional magnetic resonance imaging (fMRI) can be an instrumental tool in providing insights into neural changes when exposed to food cues over time. Therefore, in this longitudinal, pre-registered clinical trial study, mindfulness training was utilized to investigate the effect of the habituation response when exposed to persistent food stimulation. In addition, given the nature of the stimuli being presented in this study (i.e., visual-olfactory perception of high-calorie food stimuli), brain regions known to be relevant for emotion regulation, visual perception, and olfaction were employed as regions of interest (ROIs). This study could, therefore, contribute to the literature on mindfulness training as a valuable tool in addressing overeating behavior and promoting a healthier relationship with food.

## Methods

### Participants

To investigate the effects of mindfulness training on the food-related habituation response, participants who subjectively reported having the tendency to overeat when stressed were recruited via the university hospital’s mailing list and online advertisements. Participants were required to report moderate to high levels of stress, as assessed by the perceived stress scale (PSS)^37^ while expressing a tendency to engage in stress-eating and needed to fulfill the following criteria: (1) between the ages of 18 and 45 years, (2) general MRI suitability (i.e., no metal implants and not prone to claustrophobia), (3) body-mass-index (BMI) between 18 and 30 kg/m^2^, (4) no dietary restrictions (including vegetarianism or veganism)^38^, (5) no use of oral contraceptives or intrauterine devices, (6) no known untreated thyroid dysfunction, (7) no chronic respiratory diseases. All participants provided written informed consent and were given monetary compensation for their participation. This study is listed as a clinical trial on the ISRCTN registry with trial ID ISRCTN12901054 registered on 19/05/2023, was performed in accordance with the Declaration of Helsinki, and was approved by the Ethics Committee of Klinikum rechts der Isar, Technical University Munich (71/19S-SR). Participants were provided with a comprehensive information sheet detailing the study’s purpose, procedures, risks, and benefits. They also completed a checklist confirming eligibility and provided written informed consent, agreeing to specified terms regarding participation, MRI safety, and incidental findings. Participants were explicitly informed of their right to withdraw from the study at any time without any disadvantages.

### Procedure

This study was designed as a pseudo-randomized, active-control trial to investigate the effects of mindfulness meditation training (MMT) on stress-eating behavior and its neural correlates. Participants were allocated to either the MMT condition or the active control, health training (HT) condition (single-blinded, subject only) using a pseudo-randomized approach, ensuring a balanced distribution of key demographic and baseline variables such as age, gender, and stress-eating tendencies. The randomization sequence was generated using a computerized tool and implemented by an independent researcher to minimize bias. This method ensured that group sizes were approximately equal and that the risk of confounding factors was reduced. All participants underwent MRI, and psychometric testing evaluating perceived mindfulness, stress, emotional eating, food cravings, dietary restraint, and the assessment of body weight before and after completing the intervention (the detailed results of which are reported in Torske, 2024)^24^. All measures were acquired after a subjectively stressful day and participants were instructed to abstain from eating 5 h before their scheduled MRI measure. The training programs for both conditions were accessible via an online platform and consisted of 31 daily 15-min sessions. To promote training adherence, reminders were sent to participants by email.

In the MMT, participants were provided with a detailed introduction to the theoretical framework of MMT while additionally guiding participants through the daily meditation exercises via video or audio clips. Written instructions emphasized the relationship between MMT and eating behavior, encouraging participants to engage more mindfully with food. The HT condition was designed to imitate the format of the MMT and provided participants with informative health-related video and audio clip excerpts from popular science broadcasting networks in Germany. Topics in the HT did not include any information pertaining to MMT, eating behavior, or nutrition. These materials were intentionally selected to avoid content related to mindfulness or eating behaviors, ensuring that the control intervention served as a distinct comparison while maintaining comparable engagement and duration. For a detailed description of the training content, please see Table S1. Participants were required to complete at least 27 training sessions to be included in the final analysis.

**Fig. 1:**
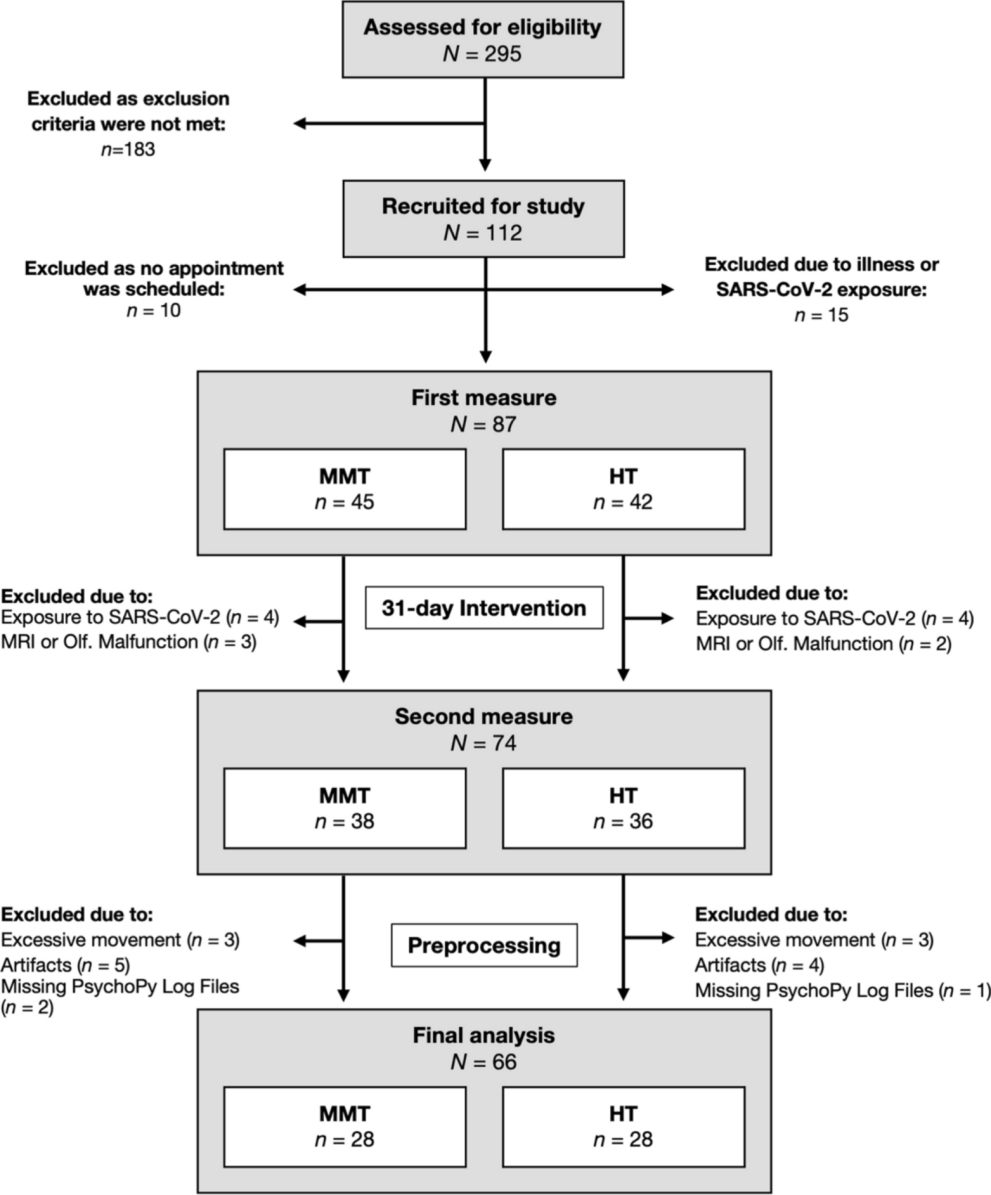
Data acquisition process (June 2019–June 2021).

295 participants were assessed for eligibility and 112 participants fulfilled the inclusion criteria. 87 participants completed the first MRI measure, and 74 participants were also available for the second MRI measure. After preprocessing, data from 56 participants (28 female) were included in the final analysis that is in accordance with our a priori sample size calculation (see Supplementary Material). Data acquisition occurred between June 2019 and June 2021 and is depicted in Fig. 1.

To verify the pseudo-randomization process, final samples were compared for demographic characteristics, measuring intervals, and an average number of sessions completed using *t*-tests for independent samples or chi-square tests, respectively.

### Functional imaging task

**Fig. 2:**
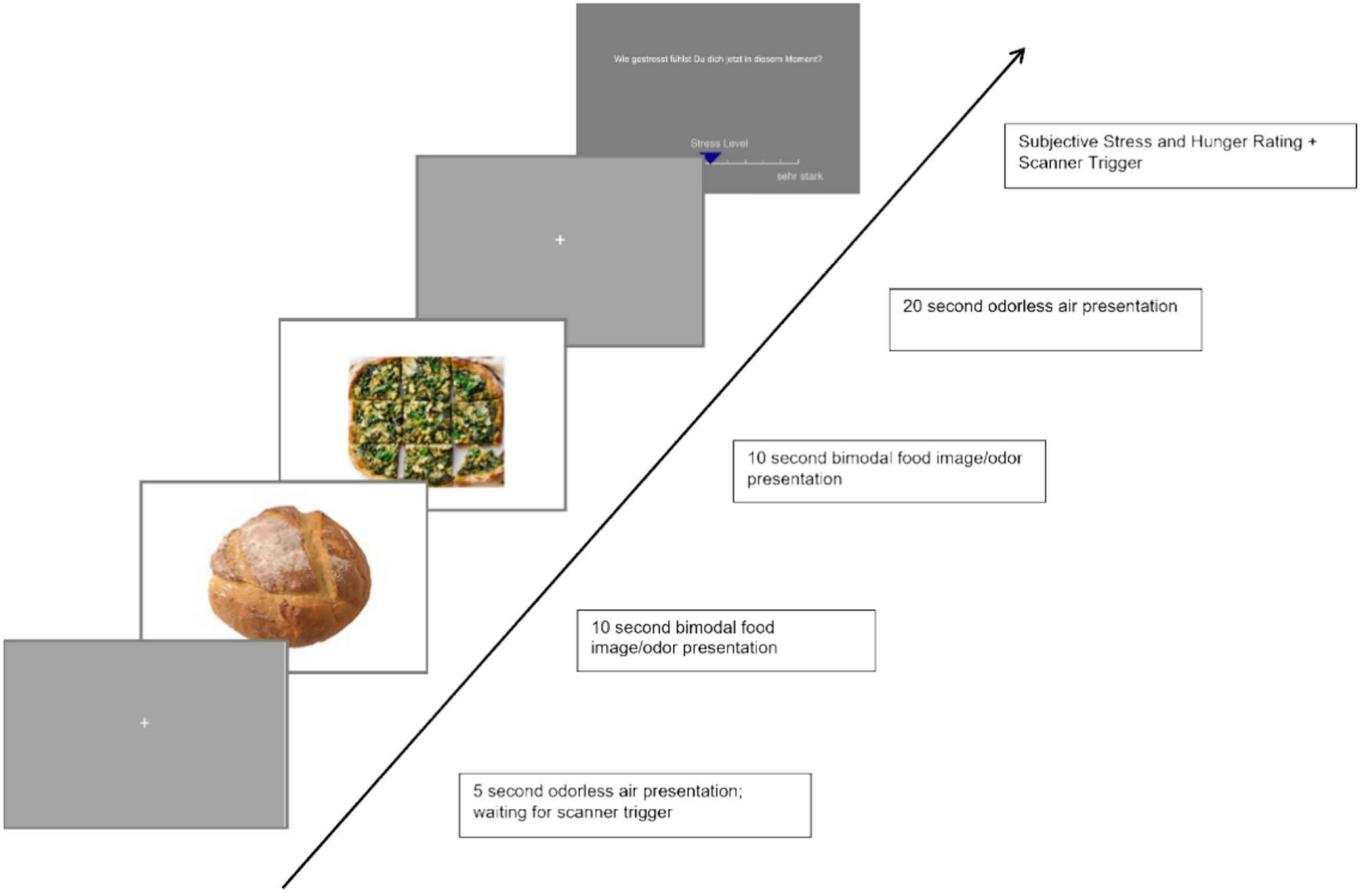
The experimental paradigm of the study.

During the fMRI sequence, the odors were systematically presented to the participants, during which two food odors and their corresponding images were pseudo-randomly (to ensure the same stimulus was not presented twice) presented to the participants (10 s) in direct succession. Following the food stimulus presentation, an odorless air condition (20 s) was presented. One block contained six bimodal food (BF) conditions (two food stimuli presentations per condition) that were each followed by an odorless air condition presented together with a fixation cross. At the end of the block, stress and hunger ratings were acquired. This sequence was repeated six times (see Fig. 2 for a graphical representation of the experimental design).

### Behavioral analysis

To analyze initial hunger and stress ratings, the respective ratings (extracted from block 1) were evaluated with mixed ANOVAs with the within-subject factor session and the between-subject factor group. We used the R package performance version 0.10.4 to test for homogeneity of variances via Levens’s Test (hunger *p* = 0.862, stress *p* = 0.072 ), sphericity (*p*> 0.999), and normality of residuals via Q-Q-Plots. All the assumptions were met. To investigate changes over time, ratings per time point were correlated with every time point utilizing repeated measurement (rm) correlation, implemented in the R-package rmcorr, version 0.5.4^39,40^. To compare whether the correlations significantly differ between the groups and between the sessions, the R-package cocor, version 1.1-4^41^ was utilized. We used the R package gglm (version 1.0.3) to analyze model diagnostics for rm correlations. Diagnostic plots revealed no severe violations of assumptions for linearity, homoscedasticity, or influential cases. While Q-Q plots occasionally showed a tailed distribution, RM correlations are robust to such deviations.

### Functional imaging parameters

All MR imaging was performed on a 3 Tesla Philips Scanner, equipped with a 32-channel head coil at Klinikum rechts der Isar, Dept. of Neuroradiology, Munich, Germany. Whole brain functional scans, with 63 AC-PC axial slices (0.2 mm interslice gap) were acquired using a T2*-weighted 2D single shot gradient echo planar (GE-EPI) sequence, MB Factor 2, TE 30 ms, TR 2300 ms, flip angle 70, FOV 192 × 192 × 136.2 mm^3^, and an 8 mm^3^ isotropic voxel resolution. The total fMRI acquisition time was 26 min. Additional high-resolution T1-weighted anatomical images were acquired using an MPRAGE sequence with the following scanning parameters: TR 11 ms, TE 5.2 ms and flip angle 8°. 230 axial AC-PC slices encompassing a 384 × 384 matrix of 0.7 × 0.7 × 0.7 mm^3^ large isotropic voxels were obtained. All anatomical images underwent clinical inspection by a neuroradiological specialist to detect possible structural pathologies.

### Preprocessing

Preprocessing of the functional images to analyze the block design experiment was conducted using Statistical Parametric Mapping (SPM12, The Wellcome Centre for Human Neuroimaging, London, UK). Participants with a framewise displacement (FDmean > 0.25) were excluded^42,43^. The pipeline to preprocess the data was as follows: Realignment to the mean functional imaging and unwarping of fMRI time-series, co-registration of anatomical MRI to mean functional image, segmentation of anatomical images, normalization, normalization to MNI space. The resulting functional images were spatially smoothed with a 4 × 4 × 4 mm full-width-at-half maximum (FWHM) Gaussian kernel. The scanning protocol defined a TR of 2.3 s and had a multiband factor of 2. This allows for the simultaneous acquisition of multiple slices within a TR. This therefore reduces the temporal difference between individual slices. Therefore, to limit data manipulation no slice time correction was performed.

### First level analysis

Again, one fMRI session consisted of six blocks, each with 6 BF presentations. At the end of each block, stress and hunger ratings were acquired. This sequence was repeated six times. To analyze the data, for each block, one BF regressor (á 20 s) was extracted as well as one air regressor (20 s), including the six respective onsets. Additionally, one regressor for all six stress ratings (duration of 0 s) and one regressor for all six hunger ratings (duration of 0 s) were created. These 14 regressors were convolved with a canonical hemodynamic response function (HRF). A high-pass filter with a cut-off at 128 s was also applied. Subject-specific movement parameters were added as regressors of no interest.

All BF conditions were contrasted against all odorless air conditions (overall contrast of interest) and were also contrasted per block (BF vs. odorless air).

### Second level analysis

To analyze whether the experimental design could effectively stimulate brain areas relevant to food image and odor processing and to analyze group and session differences, the subjects’ overall contrasts of interest were entered into a full factorial model with the factors group (HT vs. MMT) and session (session 1 vs. session 2). We report results that are Family-Wise Error (FWE)-corrected for whole-brain comparison with a threshold of *p* < .05.

### Correlation analysis within ROIs

To analyze whether the brain activation in sensory areas changes within a session as well as to observe the effect of the intervention, subject-specific ROIs were created using the leave-one-subject-out (loso) method for an unbiased approach. Therefore, per subject, a second-level design was created that included all first-level overall contrasts, excluding this subject’s, which was then explicitly masked to extract modality-dependent brain areas. To analyze brain activity in odor-related brain areas, the ALE mask for food odors^44^ was used, which includes brain areas such as the piriform cortex, amygdala, and insula. For activation in visual brain areas, a ROI generated with the neuromorphometrics atlas integrated in SPM covering the left and right occipital fusiform gyrus was used. The subject-specific ROI included all voxels in the following second-level t-contrast that survived FWE correction with a threshold of *p*< .05. For the emotion-regulating brain areas, a predefined ROI using the MAG1 mask from^45^ was used. Within each ROI, the BF beta-values from this subject’s first-level analysis were extracted and averaged per time point per block.

To investigate whether there is a change in brain activation within a session, the extracted, averaged mean beta values for BF per time point were correlated with each time point. These correlations were then compared with the correlations between sessions and groups analog to the above (see behavioral analysis).

## Results

### Behavioral results

The initial stress and hunger ratings obtained within the MRI scanner throughout the duration of the scanning session did not differ significantly between the two sessions (stress: F(1, 40) = 0.037, *p* = .849, hunger: F(1, 42) = 0.337, *p* = .565) and the groups (stress: F(1, 40) = 0.069, *p* = .795, hunger: F(1, 42) = 0.571, p = .454). Additionally, there were no significant interaction effects (stress: F(1, 40) = 0.920, *p* = .343 , hunger: F(1, 42) = 3.626, *p* = .064). These results indicate that the intervention training elicited no differences between groups across time.

### Change in hunger ratings across the experiment

**Fig. 3:**
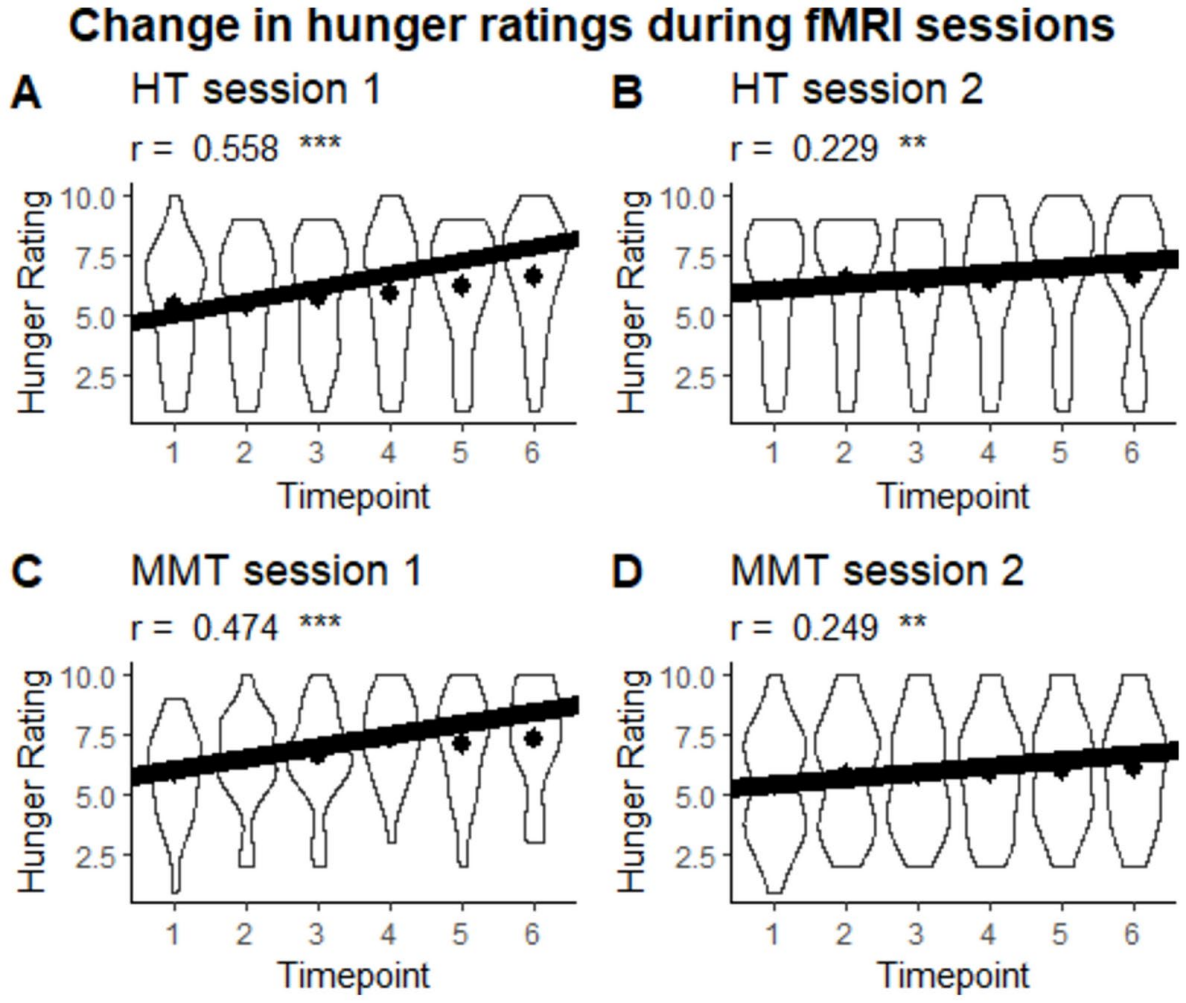
Violin plot of the change in hunger ratings during fMRI sessions per session and group. Dots represent the mean. ***p < .001, **p < .01.

While no differences in hunger ratings were detected between groups at session 2, subsequent correlation analyses were conducted in an effort to observe the habituation response. For the correlation analyses pertaining to hunger ratings, a significant positive correlation between the time point and hunger rating was observed for the HT condition in session 1 (r = 0.558, CI = [0.421, 0.670], *p* < .001, effective N = 121) and session 2 (r = 0.229, CI = [0.061, 0.384], *p* = .008, effective N = 133) as well as in MMT condition in session 1 (r = 0.474, CI = [0.324, 0.600], *p* < .001, effective N = 123) and session 2 (r = 0.249, CI = [0.081, 0.402], *p* = .004, effective N = 132). No group differences were observed in session 1 (Fisher’s z = -0.886, *p* = .376) and session 2 (Fisher’s z = 0.169, *p* = .866). However, we were able to observe session differences within the HT condition (Pearson and Filon’s z = 3.408, *p* < .001) and the MMT condition (Pearson and Filon’s z = 2.400, *p* = .016) (see Fig. 3).

### Change in stress ratings across the experiment

**Fig. 4:**
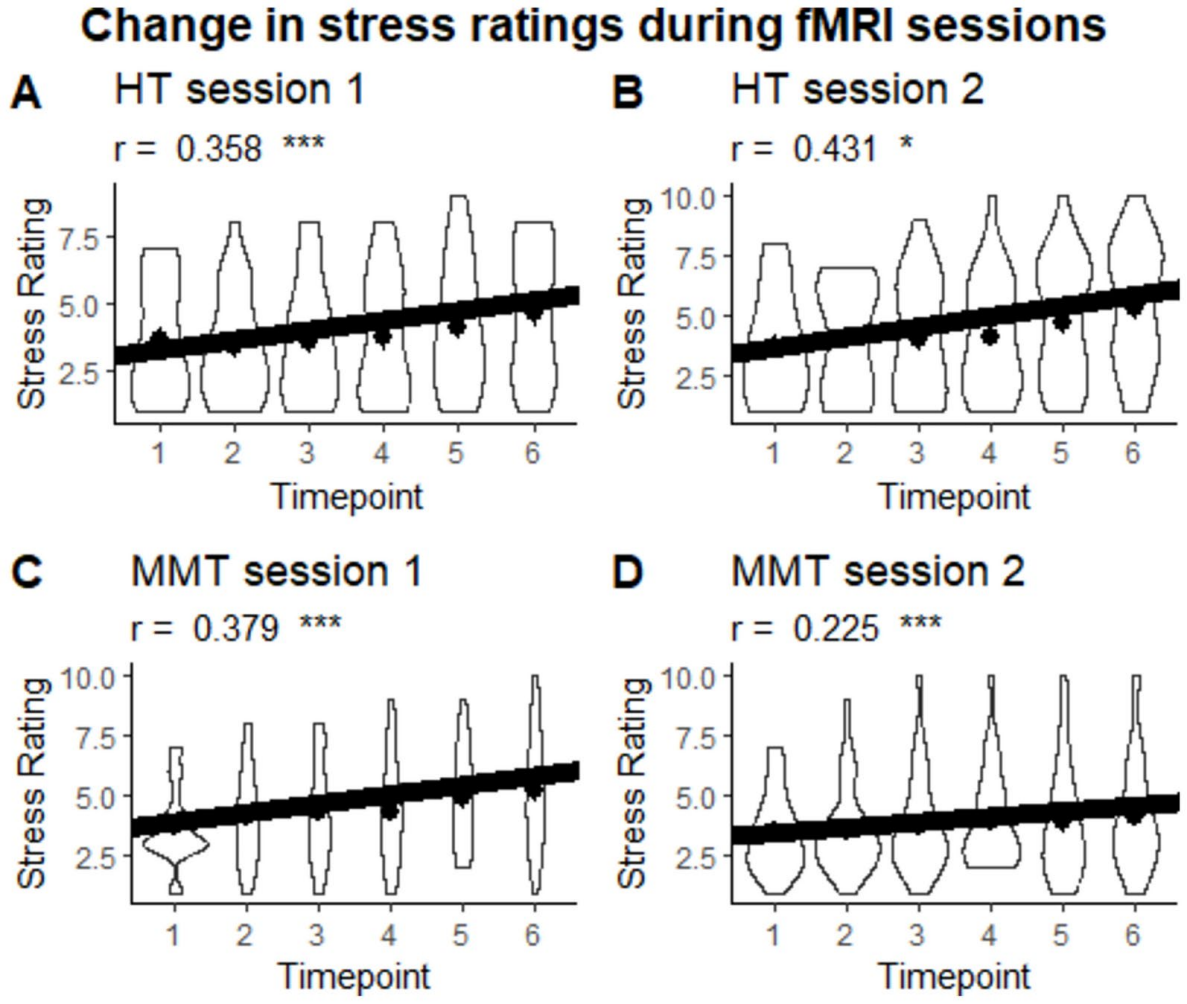
Violin plot of the change in stress ratings during fMRI sessions per session and group. Dots represent the mean.. ***p < .001, **p < .01, *p < .05.

In addition, correlation analyses were conducted to observe changes in stress ratings across the experiment. The results of these analyses demonstrate significant positive correlations in the HT condition in session 1 (r = 0.358, CI = [0.194, 0.503], *p* < .001, effective N = 123) and session 2 (r = 0.431, CI = [0.278, 0.562], *p* < .001, effective N = 128) as well as in the MMT condition in session 1 (r = 0.379, CI = [0.217, 0.521], *p* < .001, effective N = 123) and session 2 (r = 0.225, CI = [0.055, 0.382], *p* = .010, effective N = 131). No group differences exist for session 1 (Fisher’s z = 0.186., *p* = .852) and session 2 (Fisher’s z = -1.846, *p* = .065) as well as no significant session difference for the HT condition (Pearson and Filon’s z = -0.809, *p* = .418) and the MMT condition (Pearson and Filon’s z = 1.402, *p* = .161) were observed (see Fig. 4).

Those analyses indicate that in both groups hunger and stress ratings increased across the experimental sessions.

### Brain activation across sessions and groups

**Fig. 5:**
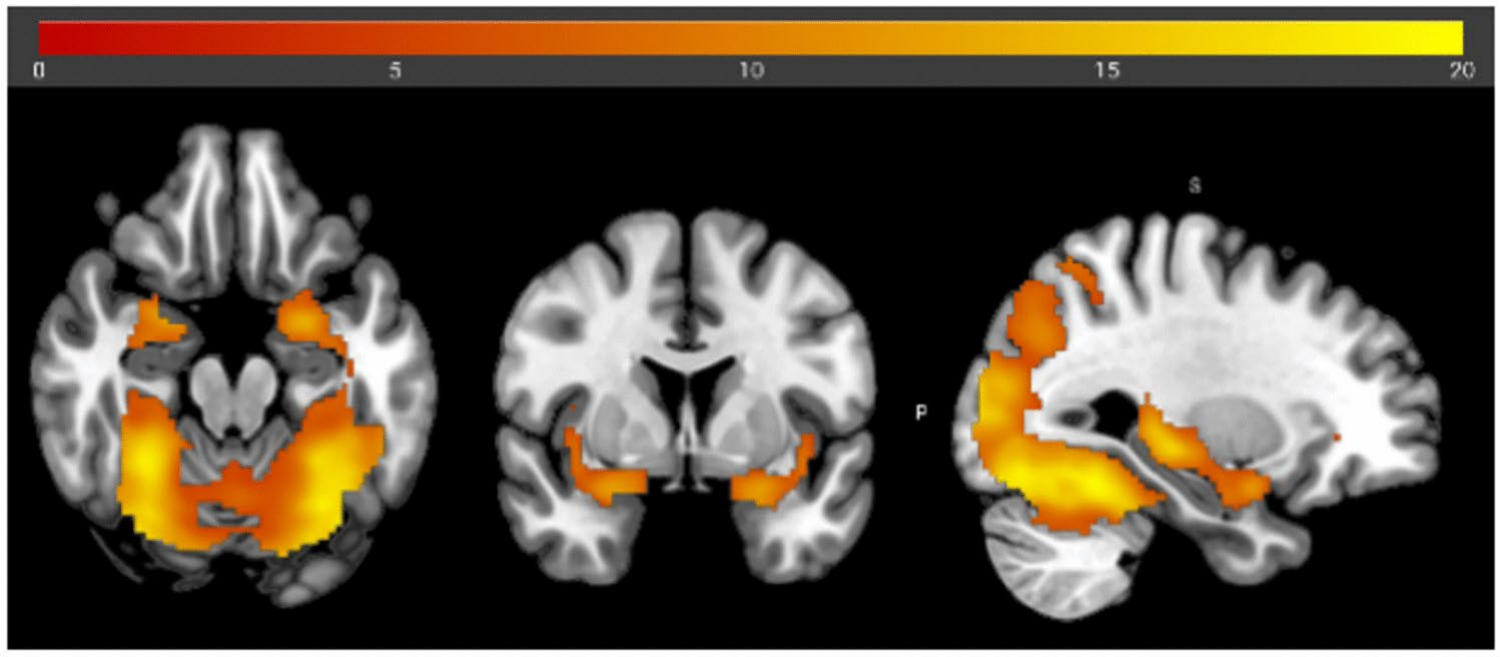
Brain activation during bimodal food image/odor presentation versus odorless air condition with an FWE threshold of.05. The coordinates of the center of the crosshair are^1,25,34^.

To determine the experiment’s effectiveness, bimodal food presentations were contrasted against air (positive effect of condition in the ANOVA). This analysis revealed bilateral activations in visual brain areas, in particular the lingual gyrus and occipital gyrus, as well as in olfactory brain areas, in particular the amygdala and piriform cortex (see Fig. 5 and Suppl. Table S2). However, there was neither a significant main effect of time nor of group, as well as no significant interaction effect. In addition, a full factorial, rm ANOVA was conducted, which resulted in no significant interactions.

### Brain activation within sessions

#### Olfactory brain areas

**Fig. 6:**
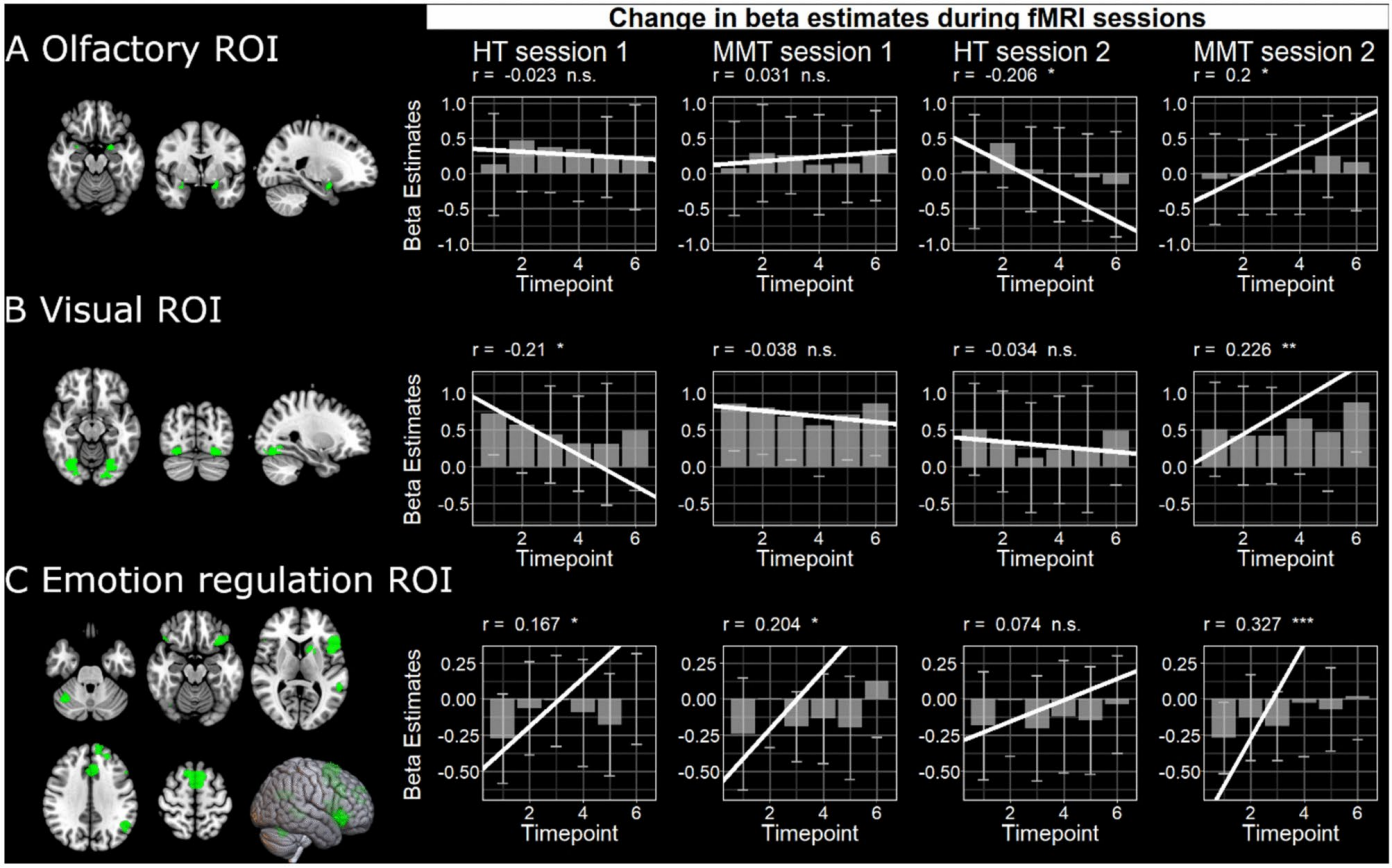
Examples of subjects-specific ROIs for olfactory and visual brain areas as well as the emotion regulation ROI. We show the mean and standard deviation of stimulus activation in these ROIs as well as the rm correlation between activity and the respective brain areas and timepoint per session and group.

To observe the effects of MMT on brain areas pertaining to olfaction during an experimental session, bilateral clusters (*p*< .05 FWE) survived for all loso-second level contrasts that were masked exclusively with the food odor mask^44^. We were successfully able to observe significant correlations between the activation of olfactory brain areas and the study duration, i.e. from timepoint 1–6, in session 2, with significant differences between the groups (see Table 1 and Fig. 6A).

**Table 1:** Rm correlation between block number (time) and beta estimated within olfactory ROI.

Group	Session	N (eff.)	r [95% CI]	*p*	Within-group comparison (session 1 vs. session 2)	
					z	*p*
HT	1	141	− 0.023 [− 0.188, 0.143]	.787	1.563	.118
HT	2	141	− 0.206 [− 0.359, − 0.042]	.014 *		
MMT	1	141	0.031 [− 0.135, 0.195]	.714	− 1.469	.142
MMT	2	141	0.200 [0.035, 0.353	.018 *		

Between-group comparison (HT vs. MMT). Session 1: Fisher’s z = 0.449, *p* = .654, Session 2: Fisher’ z = 3.417, *p* < .001**

**p < .01, (*) trend towards statistical significance a Pearson and Filson’s z.

### Visual brain areas

The previous correlation analysis was repeated for the visual cortex, and the significant bilateral clusters (*p* < .05 FWE) survived for all loso-second level contrasts. Within the MMT group, there was a significant difference between the sessions. Additionally, there was a significant group difference in session 2 (see Table 2 and Fig. 6B).

**Table 2:** Rm correlation between block number (time) and beta estimated within visual ROI.

Group	Session	N (eff.)	r [95% CI]	*p*	Within-group comparison (session 1 vs. session 2)	
					Pearson and Filon’s z	*p*
HT	1	141	− 0.210 [− 0.363, − 0.046]	.012 *	− 1.654	.098
HT	2	141	− 0.034 [− 0.200, 0.132]	.692		
MMT	1	141	− 0.038 [− 0.202, 0.129]	.658	− 2.303	.021 *
MMT	2	141	0.226 [0.063, 0.377]	.007 **		

Between-group comparison (HT vs MMT). Session 1: Fisher’s z = 1.459, *p* = .145. Session 2: Fisher’ z = 2.189, *p* = .029*

***p < .001, **p < .01, *p < .05, (*) trend towards statistical significance.

### Emotion regulating brain areas

Lastly, the correlation of brain activity in brain areas responsible for emotion regulation revealed a significant group difference at session 2 (see Table 3 and Fig. 6C).

**Table 3:** Rm correlation between block number (time) and beta estimated within emotion regualtion ROI.

Group	Session	N (eff.)	r [95% CI]	*p*	Within-group comparison (session 1 vs. session 2)	
					Pearson and Filon’s z	*p*
HT	1	141	0.167 [0.001, 0.323]	.048 *	0.835	.404
HT	2	141	0.074 [− 0.092, 0.237]	.382		
MMT	1	141	0.204 [0.040, 0.357]	.015 *	− 1.158	.247
MMT	2	141	0.327 [0.171, 0.467]	< .001 ***		

Between-group comparison (HT vs. MMT). Session 1: Fisher’s z = 0.317, *p* = .751. Session 2: Fisher’ z = 2.201, *p* = .028*

**p < .01, (*) trend towards statistical significance.

## Discussion

The findings of this study, in which we examined the influence of MMT on the habituation response to persistent food stimulus presentation, reveal several noteworthy results. First and foremost, to explore the impact of MMT on the neural level, we needed to determine whether our experimental design could effectively induce neural responses to the olfactory and visual presentation of food stimulation. To this end, by contrasting the odorous vs. baseline conditions, we were able to determine that our experimental design elicits significant bilateral activations in visual, frontal, and olfactory brain areas essential in the processing of food stimulation, exhibiting similar activations previously seen in the literature^44,46^. Neural activity at session 1 did not statistically differ which serves as an effective baseline to determine the effects of the intervention strategies.

Despite this comparable baseline, our results were unable to demonstrate a group (MMT vs. HT) by time (session 1 vs. session 2) interaction effect when looking at the odorous stimulation vs. baseline contrast analysis. These results indicate that, on the whole-brain level investigated via the GLM, individuals of MMT group did not show a differential response to food stimulation presentation in comparison to the HT group at session 2. However, as we were specifically interested in exploring MMT’s effect on the habituation response, further analyses were conducted to determine whether individuals of the MMT group and their perception of olfactory and visual food stimuli changed over the duration of the scanning session upon completing the training. We, therefore, specifically investigated the neural activity of brain areas attributed to olfactory and visual processing as well as emotion regulation to observe MMT’s effect on sensory food stimulation over time. Interestingly, despite the lack of an interaction effect when comparing activation across the sessions, our data were able to demonstrate that upon completing the intervention training (at session 2), the MMT and HT responded differently to food stimulation on the neural level over the duration of the scanning session. For example, at session 1, both the MMT and the HT groups demonstrated a slight reduction or no change in neural activity throughout the duration of the sensory food stimuli presentation in brain areas responsible for processing olfactory and visual stimulation. While these results could only demonstrate that participants of both the MMT and the HT groups at session 1 exhibited a slight habituation response or no change over time, we attribute the fact that we did not observe a strong habituation effect—that has previously been observed in the literature^47^—to the fact that participants were presented with a different odorous stimulus every 10–20 s, thereby not allowing ample time to achieve the habituation response observed in the literature. Interestingly, at session 2, the HT group demonstrated a significant decline in neural activity throughout the duration of the food stimuli presentation in brain areas responsible for odor processing. These results indicate that participants who completed the HT were more susceptible to the habituation response than the MMT participants. Interestingly, participants of the MMT demonstrated an increase in neural activation in brain areas relevant to odor processing, indicating that participants who underwent food-related MMT were not only able to sustain their attention to the odorous food stimuli throughout the duration of the scanning session but were also able to increase their attention and awareness to the odorous food stimuli over time, reflected in neural responsivity. While a previous study investigating the effects of mindfulness on sensory perception was only able to identify an increase in olfactory perception in a subset of participants^48^, our results contribute to these initial findings demonstrating that MMT can, in fact, have an influence on olfactory perception on the neural level.

Not only do our results suggest MMT’s influence on olfactory perception, but we were also able to demonstrate changes in the visual presentation of food stimulation. At session 2, our results demonstrate that while the HT group exhibits a significant reduction in neural activity in brain areas responsible for visual stimulus processing, which could indicate a strong habituation response, the MMT group exhibited a significant increase in brain areas responsible for the processing of visual stimuli, indicating an increase in perceptual awareness to the visual food stimulation. The impact of MMT on visual perception has previously been explored in the literature and observed similar results^46^. Our findings expand upon previous results by demonstrating the effects MMT can have on stimuli processing of the visual system. Our results, therefore, emphasize the influence MMT can have on eating behavior through its ability to influence or alter the processing of visual food stimuli.

We additionally sought to observe the effects of MMT on brain regions pertaining to emotion regulation. In this final ROI analysis, the results were also able to effectively demonstrate MMT-related changes in neural activity in brain areas attributed to emotion regulation processing. This was demonstrated through a significant increase in neural activity among participants who underwent MMT, while participants of the HT did not exhibit a comparable increase in neural activity over time. These results underscore the unique impact MMT has on the neural substrates underlying emotional processes and align with previous research demonstrating that MMT could alter prefrontal cortex activity during emotion regulation tasks as well as during rest via functional connectivity changes^49,50^. Furthermore, the observed neural changes in the MMT group may have significant implications for eating behavior, as heightened emotion regulation is intricately linked to improved self-control and decision-making in the context of food consumption^51,52^. Our findings suggest that MMT could be utilized as a targeted intervention for fostering healthier eating behaviors through its positive influence on emotion regulation.

On the neural level, our results were able to demonstrate MMT’s effect on sensory perception and the habituation response. These results add valuable insights to the existing literature, suggesting that MMT may serve as a promising avenue for cultivating mindful awareness and positively influencing sensory and emotional processing in the context of food consumption on the neural level.

Given the study’s ability to elicit changes in the neural response to food stimulation, we sought to observe whether MMT could elicit changes in perceived hunger as well as stress on the behavioral level during the scanning sessions. Initial stress and hunger ratings were similar for both MMT and HT groups, indicating baseline comparability. In session 1, both groups showed increased hunger and stress ratings. Given that this experiment sought to observe the influence MMT has on the habituation response, we hypothesized that individuals in the MMT group would experience a reduction in both stress and hunger ratings at session 2 upon completing the training. Interestingly, our results did not show significant differences after the intervention. The intriguing discrepancy between the observed changes at the neural level and the absence of corresponding behavioral effects prompts a nuanced examination of the temporal dynamics and potential limitations of our study. Additionally, the absence of significant behavioral differences may be attributable to the fact that all participants completed the study in a hungry and stressed condition, compounded by the loud, high-stress environment of the MRI scanner. Exposure to enticing food odors under these conditions may have further heightened feelings of hunger and stress, regardless of how mindfully participants engaged with the food stimuli. However, it is important to note that behavioral measures acquired outside of the scope of this research article were able to demonstrate a significant interaction between group and time on the behavioral levels when examining stress eating, emotional eating, and food cravings^24^. These results indicate the MMT was successfully able to reduce perceived stress- as well as emotional-eating tendencies, the reduction of which was simultaneously reflected in the degree of functional connectivity changes on the neural level. Therefore, despite successsful changes in neural responses to food stimuli, the in-scanner stress and hunger ratings may not have been able to effectively capture the behavioral effect observed in Torske et al.^24^.

However, this incongruity between our neural and behavioral outcomes and those observed in Torske et al.^24^, echoes the complex interplay between cognitive and neurological processes influenced by MMT. In fact, previous research has highlighted that the translation of neural changes to behavioral outcomes may not always be straightforward, and the behavioral effects of MMT may require an increase in training durations to become apparent^53–55^. In summary, while our food-related MMT did not result in significant alterations in in-scanner hunger and stress ratings compared to the HT, the fMRI results suggest MMT’s ability to induce neural changes in the responsivity to food stimuli and its ability to reduce the habituation response effect. The study, therefore, highlights potential MMT intervention-related changes on the neural level when perceiving food-stimuli, while additionally offering valuable insights into the complex interplay between mindfulness, stress, and neurobiological responses related to overeating behavior.

## Limitations

Despite the valuable insights provided by this study on the effects of MMT on the habituation response to persistent food stimulation, several limitations should be acknowledged. Firstly, despite the study’s large sample size, the findings we were seeking to observe on both the behavioral as well as the whole-brain neural levels may not have been large enough to detect the interaction effect. Therefore, future research with larger and more diverse participant groups is warranted. In addition, while our observed effects on the neuronal level could reflect broader changes in attention or engagement, these processes are inherently linked to mindfulness training and represent intended aspects of the intervention. Future studies could explore alternative control conditions to further disentangle these effects. Furthermore, the reliance on self-reported in-scanner stress and hunger ratings introduces the possibility of subjective bias and may not have fully capture nuanced changes in participants’ experiences, as was the case in the validated behavioral measured utilized in Torske et al.^24^. In addition, the absence of a long-term follow-up assessment limits our understanding of the durability of MMT-induced changes with regard to ﻿eating behavior. Therefore, future study designs should seek to incorporate more objective, standardized, and validated measures of food intake or physiological stress responses, instead of relying primarily on subjective in-scanner ratings and neuroimaging data. Finally, we did not systematically control for external factors, such as dietary habits, lifestyle changes, or concurrent stressors, which could confound the interpretation of the results. Addressing these limitations in future research would contribute to a more comprehensive understanding of the nuanced relationship between MMT, stress-related overeating behavior, and associated neurobiological mechanisms.

## Conclusion

This study sheds light on the impact of MMT on stress-related overeating behavior, revealing both notable findings and important considerations. While the MMT intervention did not significantly alter self-reported stress and hunger ratings compared to the HT, the neuroimaging results indicated MMT’s ability to induce activation-related changes on the neural level pertaining to the habituation response of food stimuli presentation. The study’s limitations, including a modest sample size, reliance on subjective measures, and a lack of long-term follow-up, should be acknowledged. Despite these limitations, the study contributes valuable insights into the complex interplay between mindfulness, stress, and neural responses related to overeating. The observed session-related variations in hunger and stress ratings underscore the dynamic nature of these factors over time. Future research with larger, more diverse samples, objective measures of eating behavior, and longer follow-up periods is warranted. Understanding the nuanced relationship between mindfulness, stress, and overeating behavior holds potential implications for developing effective interventions to promote mindful eating and mitigate stress-induced overeating in diverse populations.

## Supplementary Information

Supplementary Information.

## Data Availability

Data have been made publicly available via the Open Science Framework at https://osf.io/pf3gv/.
