# Supplementary material for: Mindfulness training decreases the habituation response to persistent food stimulation
Source: Sci Rep. 2025 Apr 25;15:14479. doi: 10.1038/s41598-025-90172-3 (PMC12032164; doi:10.1038/s41598-025-90172-3)
Supplement: Supplementary file 1 — Supplementary Information. [file 41598_2025_90172_MOESM1_ESM.docx]

**Supplementary Section:**

**Sample Size Calculation**

We conducted an a priori sample size calculation for a within-between interaction (2 groups, 2 timepoints) in a two-way repeated measures ANOVA using G*Power 3.1.9.7. Based on this calculation, to achieve a statistical power of at least .80 with a small effect size of .20—as we are not aware of directly comparable studies—and an alpha error probability of .05, the required total sample size was 52 participants.

**Table S1**

|  |  | **MMT** |  | **HT** |
| --- | --- | --- | --- | --- |
| **Session** | **Format** | **Theoretical training** | **Practical training** | **Theoretical training** |
| **1** | Video | Introduction to mindfulness | Mindful breathing A | Sleep |
| **2** | Audio | *(Practice only)* | Mindful breathing A | Chronic pain |
| **3** | Audio | *(Practice only)* | Mindful breathing A | Light exposition and health |
| **4** | Video | Arriving in presence | Mindful breathing B | Sleep disturbances |
| **5** | Audio | *(Practice only)* | Mindful breathing B | Body memory |
| **6** | Audio | *(Practice only)* | Mindful breathing B | Migraine |
| **7** | Video | Arriving in the body | Bodyscan A | Burnout |
| **8** | Audio | *(Practice only)* | Walking meditation | Equanmity |
| **9** | Audio | *(Practice only)* | Bodyscan A | Social inequality and health |
| **10** | Video | Subjectivity of perception | Bodyscan B | Sore muscles / vegan diet |
| **11** | Audio | *(Practice only)* | Walking meditation | Happiness |
| **12** | Audio | *(Practice only)* | Bodyscan B | Time perception |
| **13** | Video | Communicating mindfully | Mindful attention to body sensations | Gender-specific health |
| **14** | Audio | *(Practice only)* | Mindful attention to body sensations | Illness as language of the soul |
| **15** | Audio | *(Practice only)* | Mindful attention to body sensations | Aging |
| **16** | Video | Non-judgement | Mindful attention to body sensations | Sugar |
| **17** | Audio | *(Practice only)* | Mindful listening | Medicinal plants |
| **18** | Audio | *(Practice only)* | Mindful listening | Self-deceit |
| **19** | Video | Dealing with stress | Mindfully approaching emotions | Maintaining health |
| **20** | Audio | *(Practice only)* | Mindfully approaching emotions | Migration and health |
| **21** | Audio | *(Practice only)* | Mindfully approaching emotions | Epigenetics |
| **22** | Video | Turning towards instead of turning away | Turning towards instead of turning away | Sensible footwear |
| **23** | Audio | *(Practice only)* | Approaching unpleasant feelings | Obsessive-compulsive disorder |
| **24** | Audio | *(Practice only)* | Awareness of thinking | Self-efficacy |
| **25** | Video | Positive qualities | Loving kindness | Microorganisms |
| **26** | Audio | *(Practice only)* | Loving kindness | Cardiovascular diseases |
| **27** | Audio | *(Practice only)* | Loving kindness | Hypnotherapy |
| **28** | Video | Decentring | Open monitoring | Staying active in the office |
| **29** | Audio | *(Practice only)* | Open monitoring | Negative empathy |
| **30** | Audio | *(Practice only)* | Silent meditation | Pain perception |
| **31** | Video | Reflecting the course | Silent meditation | Physical activity |

**Table S2** Brain activation during bimodal food image/odor presentation versus odorless air condition with an FWE threshold of 0.05. We report MNI coordinates of the peaks for each cluster and subcluster. Local maxima are labelled using AAL3 (Rolls et al., 2020).

| Anatomical Region | Cluster size | x | y | z |
| --- | --- | --- | --- | --- |
| Right lingual gyrus | 24059 | 20 | -84 | -10 |
|  |  | 10 | -88 | -8 |
|  |  | -14 | -98 | -4 |
| Left inferior frontal gyrus, triangular part | 523 | -48 | 34 | 18 |
|  |  | -52 | 28 | 24 |
|  |  | -54 | 18 | 34 |
| Left postcentral gyrus | 405 | -62 | -12 | 20 |
|  |  | -62 | 8 | 12 |
|  |  | -58 | 14 | 4 |
| Right inferior frontal gyrus, triangular part | 341 | 50 | 38 | 12 |
|  |  | 54 | 32 | 22 |
| Left caudate nucleus | 307 | -6 | 26 | 6 |
|  |  | -14 | 30 | 2 |
|  |  | 2 | 24 | 4 |
| Right paracentral lobule | 127 | 6 | -40 | 66 |
|  |  | 12 | -34 | 54 |
| Left paracentral lobule | 112 | -12 | -26 | 64 |
|  |  | -20 | -22 | 60 |
| Right paracentral lobule | 96 | 12 | -24 | 68 |
|  |  | 22 | -20 | 60 |
| Left precentral gyrus | 68 | -40 | -10 | 36 |
| Left angular gyrus | 61 | -44 | -50 | 28 |
| Left precuneus | 110 | -8 | -42 | 62 |
|  |  | -12 | -32 | 52 |
| Left temporal pole: superior temporal gyrus | 34 | -48 | 16 | -12 |
| Right inferior frontal gyrus, triangular part | 27 | 30 | 32 | 0 |
| Right precentral gyrus | 23 | 46 | -6 | 38 |
| Right supramarginal gyrus | 24 | -52 | -38 | 32 |
| Right temporal pole: superior temporal gyrus | 13 | 52 | 14 | -6 |
| Left inferior parietal gyrus | 14 | -50 | -38 | 44 |
| Right caudate nucleus | 12 | 24 | -14 | 30 |
| Left lobule X of cerebellar hemisphere | 6 | -22 | -42 | -42 |
| Left anterior cingulate cortex, supracallosa | 4 | -6 | 16 | 18 |
| Right postcentral gyrus | 4 | 32 | -34 | 62 |
| Left lobule IV, V of cerebellar hemisphere | 4 | -14 | -36 | -32 |
| Left postcentral gyrus | 1 | -36 | -28 | 40 |
| Right anterior cingulate cortex, supracallosa | 2 | 6 | 4 | 26 |

**References**

Rolls, E. T., Huang, C. C., Lin, C. P., Feng, J., & Joliot, M. (2020). Automated anatomical labelling atlas 3. *Neuroimage*, *206*, 116189.
